# Poster Session I - A175 COMPARATIVE ANALYSIS OF RESIDUAL NEOPLASIA AND PROCEDURAL OUTCOMES IN CURATIVE VERSUS NON-CURATIVE COLORECTAL ENDOSCOPIC SUBMUCOSAL DISSECTION

**DOI:** 10.1093/jcag/gwaf042.175

**Published:** 2026-02-13

**Authors:** F D’Anna, N Ahmed, A Kaissi, M Rai, R Bechara

**Affiliations:** Core Internal Medicine, University of Alberta Faculty of Medicine & Dentistry, Edmonton, AB, Canada; McGill University, Montreal, QC, Canada; McGill University, Montreal, QC, Canada; Medicine, Kingston Health Sciences Centre, Kingston, ON, Canada; Medicine, Kingston Health Sciences Centre, Kingston, ON, Canada

## Abstract

**Background:**

Endoscopic submucosal dissection (ESD) achieves en bloc removal of colorectal neoplasia. Resections with positive margins, lymphovascular invasion (LVI), or deep submucosal invasion (SMI) are considered non-curative, though margin positivity alone may rarely cause recurrence in non-invasive disease.

**Aims:**

To compare baseline, procedural, and pathological characteristics of curative versus non-curative colorectal ESD in a Western tertiary center, and describe management and outcomes of R1 resections.

**Methods:**

We retrospectively analyzed colorectal ESDs (July 2016-Dec 2022). Cases were classified as curative or non-curative per standard pathological criteria. Baseline, procedural, and histologic variables were compared; R1 resections were reviewed for management and outcomes.

**Results:**

We analyzed 192 ESDs (158 curative, 34 non-curative; colon 100, rectum 92); median age 70.5 years (36–94). Most lesions were JNET 2B (82.6%). Groups did not differ by age, lesion size, or LST type. Non-curative resections were more often rectal (60.0% vs 44.3%, p = 0.038), JNET 3 (27.3% vs 0.6%, p < 0.001), and had previously been biopsied (70.6% vs 49.4%, p = 0.036). No significant between-group differences were observed in procedural metrics, en bloc resection (98.7% vs 97.1%, p = 0.445), or adverse events (3.1% vs 0%, p = 1.00). Pathology diverged: R0 was less frequent in non-curative cases (55.9% vs 96.8%, p < 0.001), adenocarcinoma was more common (64.7% vs 25.0%), and HGD less common (20.6% vs 58.2%) (both p < 0.001). Of the 34 non-curative cases, 6 proceeded to surgery with no residual disease. Fourteen were followed with surveillance (median 12 months, range 3-43) without clinical or endoscopic recurrence; 2 died of other causes before follow-up, and the remaining 12 were either awaiting follow-up or managed by their referring service.

**Conclusions:**

In this series, non-curative classification was driven by adverse histology and deep invasion rather than technical performance. The small number of true R1 resections and limited follow-up precluded disease-free survival analysis, but the absence of observed recurrence supports consideration of close surveillance in carefully selected non-curative cases after multidisciplinary review and shared decision-making

**Funding Agencies:**

None

